# A Novel Mycolactone Toxin Obtained by Biosynthetic Engineering

**DOI:** 10.1002/cbic.200700411

**Published:** 2007-09-28

**Authors:** Hui Hong, Tim Stinear, Jessica Porter, Caroline Demangel, Peter F Leadlay

**Affiliations:** a Sanger Building, Department of Biochemistry, University of Cambridge80 Tennis Court Road, Cambridge CB2 1QW, (UK) E-mail: pfL10@mole.bio.cam.ac.uk; b Department of Microbiology, Monash UniversityWellington Road, Clayton, 3800, (Australia); c Unité de Génétique Moléculaire Bactérienne, Institut Pasteur28 rue du Docteur Roux, 75724 Paris Cedex 15, (France)

**Keywords:** Buruli ulcer, cytochromes, genetic engineering, *Mycobacterium ulcerans*, mycolactones

Mycolactones are polyketide macrolide toxins produced by the emerging human pathogen *Mycobacterium ulcerans*, the causative agent of the destructive skin disease Buruli ulcer.^1^, ^2^ Mycolactone appears to be the primary virulence determinant for the infection,^2^ and purified mycolactone has potent cytotoxic, apoptotic and immunomodulatory properties.^2^–^4^

The structures of mycolactones A/B ([*M*+Na]^+^ at *m/z* 765) have been determined, and their configuration has been confirmed by chemical synthesis.^2^, ^5^–^7^ The molecules are *Z* and *E* isomers of a 12-membered macrolactone linked via an ester bond with a highly unsaturated polyketide chain (Scheme 1). Further work has revealed the heterogeneity of the toxin from different geographical sources, with structural variations in the side chain that could be traced to specific changes in the biosynthetic pathway.^8^–^11^ More recently, distinctive mycolactones with characteristically different side chains have also been isolated from the frog pathogen *Mycobacterium liflandii*^12^, ^13^ and from several mycobacterial fish pathogens^14^ (see below); this raises the question of which structural features are crucial for the several effects of mycolactone on human cells.

**Scheme 1:**
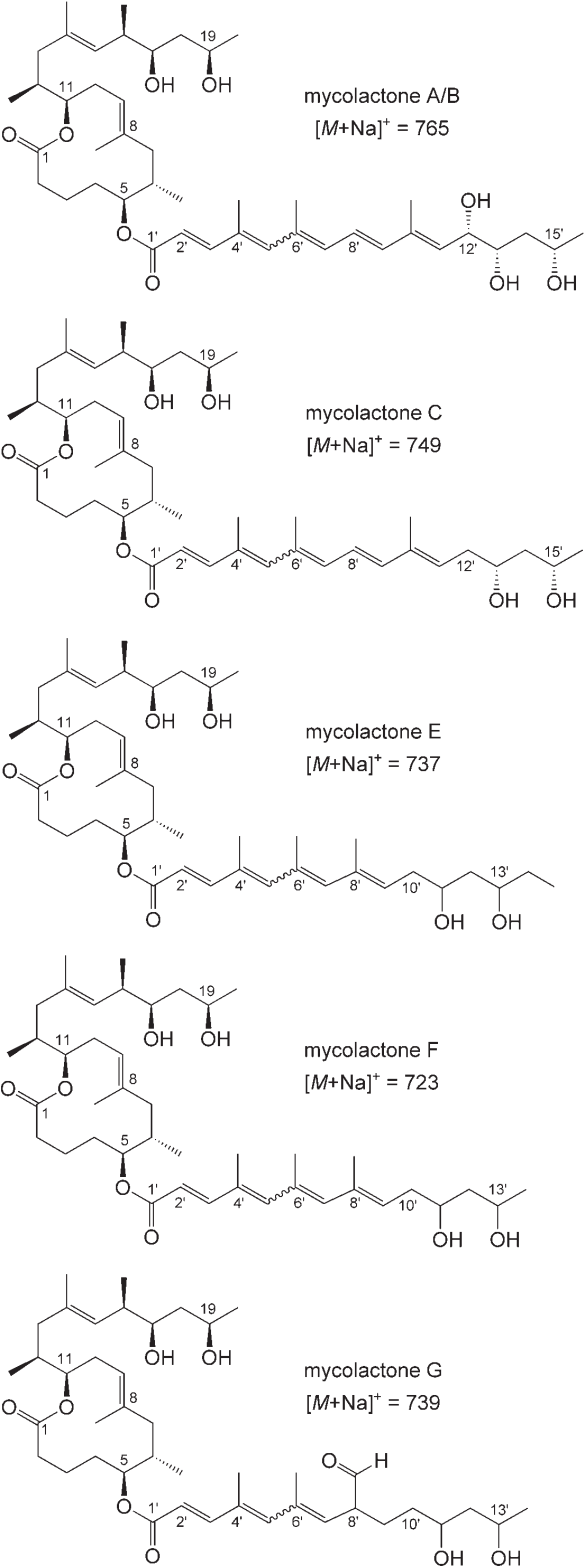
Structures of mycolactones A/B, C, E, F and G.

The genetic basis for mycolactone biosynthesis has been determined.^15^ *M. ulcerans* has a 174 kb megaplasmid harbouring three genes encoding type I modular polyketide synthases (PKS). Genes *mlsA1* and *mlsA2* together encode the PKS for production of the 12-membered core lactone, while *mlsB* encodes the PKS for the side chain. Three additional genes are present, MUP_045, MUP_038 and MUP_053. MUP_045 encodes an enzyme resembling FabH-like type III ketosynthases (KS), MUP_038 encodes a discrete thioesterase, and MUP_053 encodes a cytochrome P450 hydroxylase Cyp140A7 that is strongly implicated in the catalysis of oxidation at C-12′ of the side chain. For example, Australian strains of *M. ulcerans*, whose virulence plasmid lacks MUP_053, typically produce mycolactone C, in which the C12′ hydroxy group is missing.^16^ We wished to test whether Cyp140A7 might oxidise an alternative mycolactone substrate to give rise to additional structural diversity by biosynthetic engineering. We now report that when Cyp140A7 was expressed in the recently discovered fish pathogen *Mycobacterium marinum* DL045, a novel mycolactone (mycolactone G) was indeed produced from the engineered strain. Importantly, mycolactone G and other mycolactone variants were found to differ significantly from mycolactones A/B in their immunosuppressive activity.

We first examined the structure of the mycolactones normally produced by several recently discovered *Mycobacterium ulcerans*-like mycobacteria isolated from diseased fish.^14^, ^17^ Cell extracts from each of *M.* *marinum* DL045, *M.* *marinum* CL240299, *M.* *marinum* DL240290 and *M. pseudoshottsii* L15, were analysed by LC-MS and LC-MS/MS. These strains all produced mycolactones with *m/z* at 723 (major component; mycolactone F) and *m/z* 721 (minor component). The MS/MS spectra of *m/z* 723 and of *m/z* 721 from all the fish pathogen strains checked were identical; this suggests that these strains produced the same mycolactones. The MS/MS analysis also showed that the core lactones from these two mycolactone analogues were identical to the core of all mycolactones discovered so far. High-resolution mass analysis of the *m/z* 723 and 721 peaks provided the formulae C_42_H_68_O_8_Na and C_42_H_66_O_8_Na, respectively. By comparison with the formulae C_43_H_70_O_8_Na and C_43_H_68_O_8_Na of the mycolactone analogues recently reported from *M. liflandii*,^12^–^13^ (*m/z* 737 and 735, respectively, dubbed mycolactone E), these fish pathogen mycolactones appear to lack a methyl group. Comparison of the MS/MS spectra of *m/z* 723 and *m/z* 737 (Figure 1) revealed that fragment ions C, D and E, whose identities we have previously assigned as structures arising from the conjugated double bonds of the mycolactone side chain (C1′ to C8′),^11^ were present in both spectra. In addition, both compounds could lose a 120 Da fragment, which has been assigned as 1,3,5-trimethylbenzene, from the side chain.^13^ These data strongly suggest that the cumulated double bonds in the side chain are identical in the two molecules, and that the methyl difference lies in the distal (hydroxy) part of the side chain. Comparison of the fragment ions relating to this part of the side chain showed that instead of an observed loss of 58 and 102 Da in the MS/MS 737 spectrum (for mycolactone E) there was an observed loss of 44 and 88 Da in the MS/MS 723 spectrum. This finding clearly demonstrates that, compared to mycolactone E ([*M*+Na]^+^ at *m/z* 737) produced by the frog pathogen, the mycolactone from the fish pathogen ([*M*+Na]^+^ at *m/z* 723) has acetate instead of propionate as its starter unit. An acetate starter is typical of the mycolactones produced by the human pathogen *M. ulcerans*.^2^, ^7^, ^8^ Therefore, based on our previously published structure of mycolactone E,^13^ the structure for the mycolactone from the fish pathogens is that shown in Figure 1. The same structure has been recently proposed for this compound by Small and colleagues^14^ and named there as mycolactone F. Both mycolactone F from the fish pathogen and mycolactone E from the frog pathogen have one less CH_2_=CH_2_ unit in the side chain than mycolactones A/B from *M. ulcerans*. The shorter side chain might arise if an entire extension module (module 4) were missing from the PKS MLSB, which is responsible for synthesising the side chain of mycolactone A/B,^15^ but this remains to be established.

**Figure 1:**
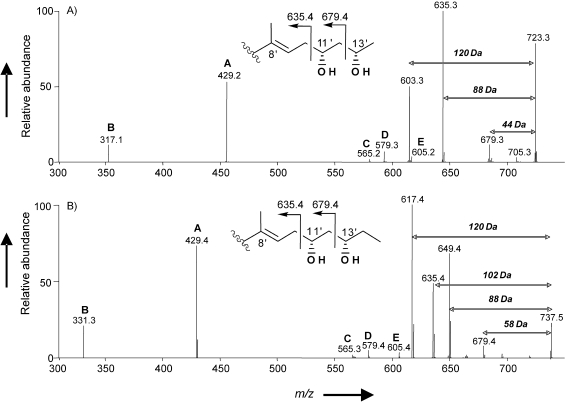
MS/MS spectrum of A) mycolactone F at *m/z* 723 (from the fish pathogen, *M. marinum* DL045) and B) mycolactone E at *m/z* 737 (from the frog pathogen, *M. liflandii*).

The production of mycolactone F by the fish pathogen *M.* *marinum* DL045 is not accompanied by any significant amount of the hydroxylated form (expected [*M*+Na]^+^ at *m/z* 739), which would be produced if a cytochrome P450 acted on the side chain of mycolactone F. We therefore tested the ability of the cytochrome P450 Cyp140A7 encoded by MUP_053 from *M. ulcerans* to bioconvert mycolactone F into a novel oxidised mycolactone, when this gene was cloned into *M. marinum* DL045. LC-MS analysis of cell pellet extracts from the engineered strain clearly showed that coexpression of the heterologous cytochrome P450 gave rise to new peaks at *m/z* 739, 16 mass units higher than mycolactone F (*m/z* 723), as shown in Figure 2. LC-MS/MS analysis at *m/z* 739 resulted in a typical mycolactone MS/MS spectrum, with characteristic fragment ions at *m/z* 429 (ion A) and *m/z* 333 (ion B), corresponding to the core lactone and the side chain, respectively (Figure 3). High-resolution mass analysis confirmed that this novel mycolactone is indeed the oxidised product of mycolactone F (designated as mycolactone G in Table 1).

**Figure 2:**
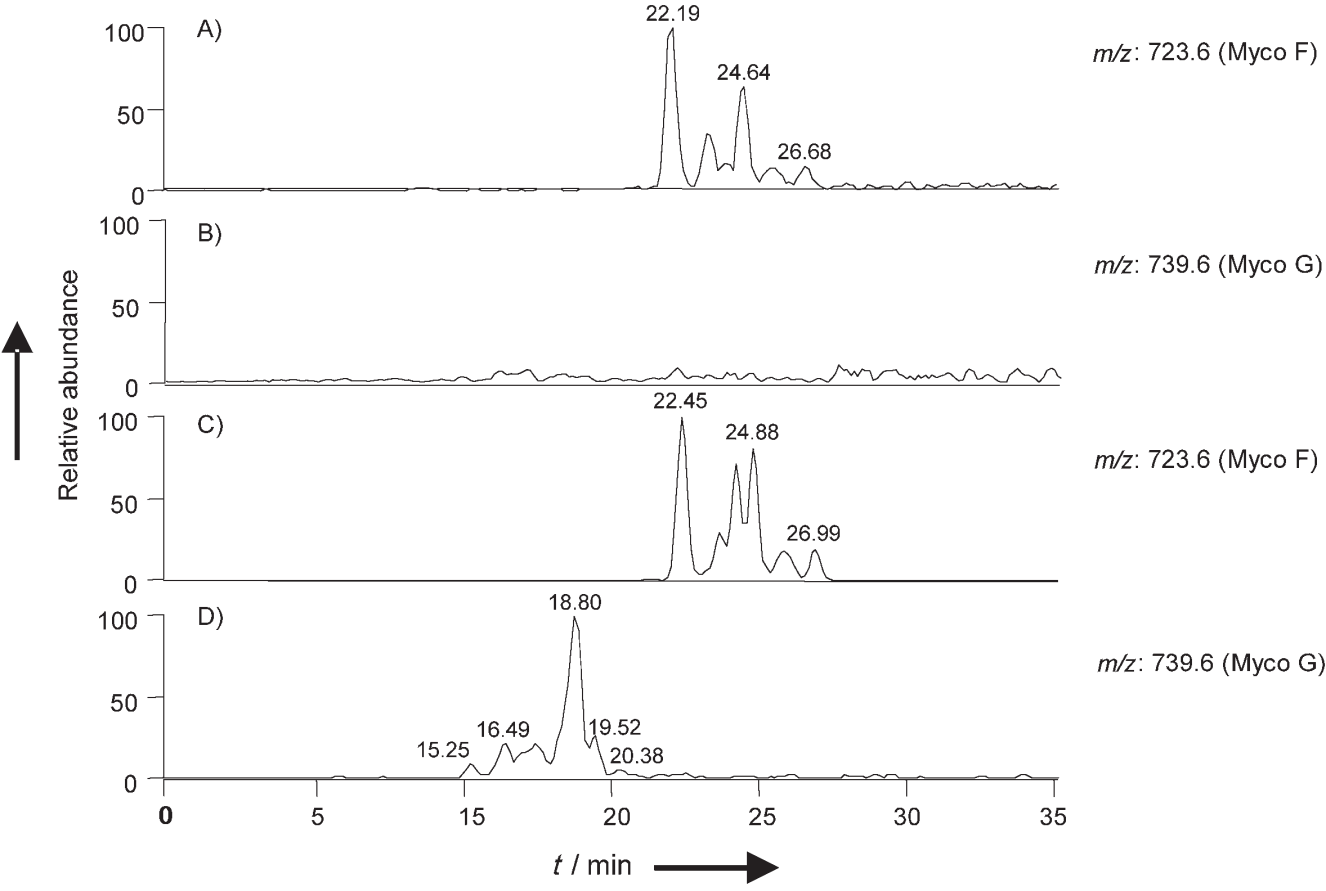
LC-MS analysis of lipid extracts from A) and B) *M. marinum* DL045; C) and D) *M. marinum* DL045 in which cytochrome P450 Cyp140A7 is expressed. The conversion to mycolactone G is signalled by its [*M*+Na]^+^ at *m/z* 739 compared to mycolactone F ([*M*+Na]^+^ at *m/z* 723).

**Figure 3:**
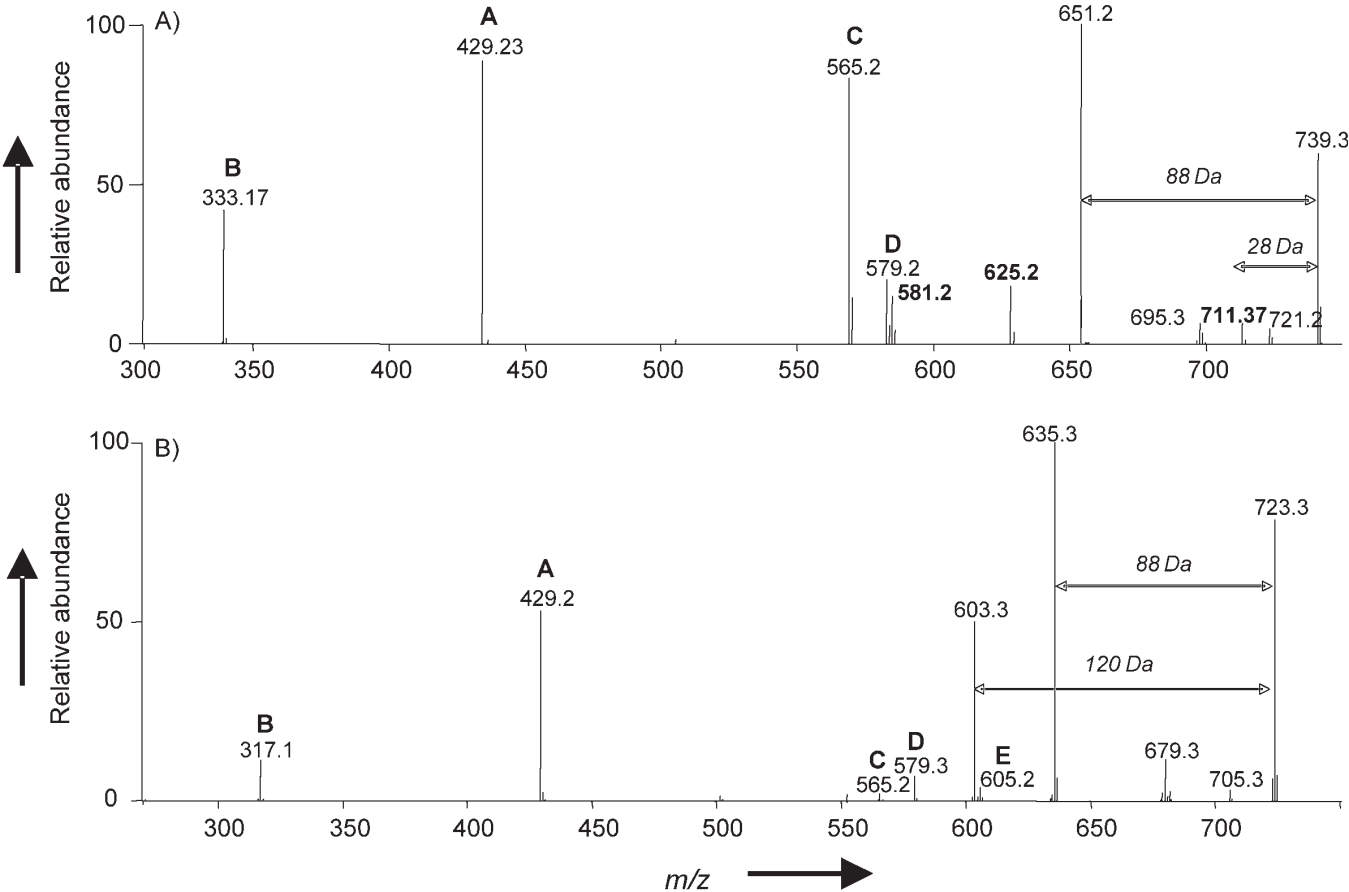
MS/MS spectrum of A) mycolactone G at *m/z* 739 and B) mycolactone F at *m/z* 723.

**Table 1:** Comparison of molecular formulae and number of exchangeable protons in mycolactone F from fish pathogen and the engineered mycolactone G.

Metabolite I.D.	Metabolite [*M*+Na]^+^	Formula	Observed mass	Error (ppm)	*n*[a]
Mycolactone F (from *M. marinum* DL045)	723	C_42_H_68_O_8_Na	723.4800	1.6	4
Mycolactone G (from *M. marinum* DL045 plus MUP_053)	739	C_42_H_68_O_9_Na	739.4765	0.5	4

[a] Number of deuterons after exchange.

To identify the exact position of oxidation, deuterium exchange and periodate oxidation experiments were performed on HPLC-purified mycolactone G. If MUP_053 acted in *M.* *marinum* DL045 exactly as in *M. ulcerans* Agy99, a hydroxy group would be introduced at C10′ of mycolactone F, the position equivalent to the C12′ in mycolactone A/B. Then, mycolactone G should, like mycolactone A/B, have five exchangeable hydrogens, one more than its precursor mycolactone F, and it should be cleavable by periodate. However, no periodate cleavage was observed on mycolactone G under conditions under which mycolactone A/B was fully converted to its aldehyde oxidation product at *m/z* 675 ([*M*+Na]^+^). Also, when mycolactone G was treated with deuterated methanol, only four instead of five protons were exchanged by deuterium. Therefore mycolactone G does not bear a hydroxy group at C10′. Detailed comparisons between the MS/MS spectra of mycolactone G (*m/z* 739) and of mycolactone F (*m/z* 723; Figure 3) showed that both peaks gave rise to ion C (at *m/z* 565) and ion D (at *m/z* 579); this suggests that the C1′–C7′ part of the side chain of mycolactone G is the same as that of mycolactone F.^11^, ^13^ In contrast, the 120 Da fragment, which corresponds to the loss of 1,3,5-trimethylbenzene from the side chain, was missing in the MS/MS of mycolactone G; this suggests that the four cumulated double bonds required to permit loss of substituted benzene from the side chain^13^ are not present. This change also resulted in the absence of fragment ion E at *m/z* 605.^13^ Compared to the MS/MS spectrum of mycolactone F, the spectrum of mycolactone G (Figure 3) also showed three new fragment ions at *m/z* 581, 625 and 711. High-resolution MS/MS analysis on mycolactone G (*m/z* 739; see the Supporting Information) showed that it could lose a CO molecule to form the fragment ion at *m/z* 711, a strong indication of an aldehyde group in the side chain. Also, MS^3^ on the fragment ion at *m/z* 651, arising from the loss of (C_4_H_8_O_2_, β-OH-*n*-butyraldehyde^13^), showed that mycolactone G could lose C_4_H_8_O_2_ from either the core or the side chain, thus indicating that (like mycolactone F) mycolactone G contains the 1,3-dihydroxyl C4 unit (C11′-C14′) intact at the end of the side chain. Therefore, the only possible position that could be oxidised and converted to an aldehyde group is the methyl branch at C8′. An aldehyde at this position also fits with the observation of two unique fragment ions at *m/z* 581 and 625 in the MS/MS spectrum of 739, produced via a hemiacetal intermediate (see Supporting Information).

To confirm the novel structure of mycolactone G, the purified compound was treated either with NaBH_4_ or with *O*-(2,3,4,5,6-pentafluorobenzyl)hydroxylamine (PFBHA). Upon treatment with NaBH_4_, a peak at *m/z* 741 was observed that coeluted with the remaining unreacted mycolactone G (*m/z* 739; data not shown). Fractions containing *m/z* 741 were collected, lyophilised and analysed by using Fourier transform ion cyclotron resonance (FTICR) mass spectrometry. The FTICR data confirmed that *m/z* 741 was indeed the reduced form of *m/z* 739 (see Supporting Information). The lyophilised sample was also treated with deuterated methanol, upon which, as expected, the reduced mycolactone G at *m/z* 741 shifted to *m/z* 746 by exchanging five deuterons, while the remaining unreacted mycolactone G at *m/z* 739 shifted to *m/z* 743 with four exchanged deuterons (see the Supporting Information). When purified mycolactone G was treated with PFBHA, a pentafluorobenzyloxime (PFBO) derivative at *m/z* 934 was observed by LC-MS; its formula was further confirmed by high-resolution MS analysis (see the Supporting Information). On the basis of this evidence taken together, we propose that mycolactone G has the structure shown in Scheme 1. The relative and absolute configurations of mycolactones A/B^5^–^7^ and C^18^ have been established by total synthesis. For mycolactones E, F and G this remains to be established. We initially expected that hydroxylation by the heterologously expressed cytochrome P450 would occur on mycolactone F at C10′, the position equivalent to C12′ in mycolactone C (Scheme 1). Intriguingly, the hydroxylation of this substrate was found to take place at the neighbouring methyl branch at C8′, presumably because of small alterations in the geometry of the enzyme–substrate interaction.

The biological activities of mycolactone variants were compared in assays of cell cytotoxicity and cytokine production by human lymphocytes. None of the variants induced detectable levels of apoptosis, as determined by phosphatidylserine exposure, in Jurkat T-cells incubated with up to 1 μg mL^−1^ mycolactone for 24 h (data not shown). However, when T-cells were activated with phorbol 12-myristate-3-acetate (PMA)/ionomycin after exposure to mycolactone, the production of interleukin (IL)-2 was repressed in a dose-dependent way by each of the mycolactones. However, the mycolactone structural variants differed markedly in their immunosuppressive activity. Mycolactones A/B proved to be the most potent inhibitor of IL-2 production, thus suggesting that the presence of a hydroxyl group on C12′ is critical for immunosuppression. Mycolactones C, E and F showed lower inhibitory effects, while mycolactone G was the least active of all (Figure 4).

**Figure 4:**
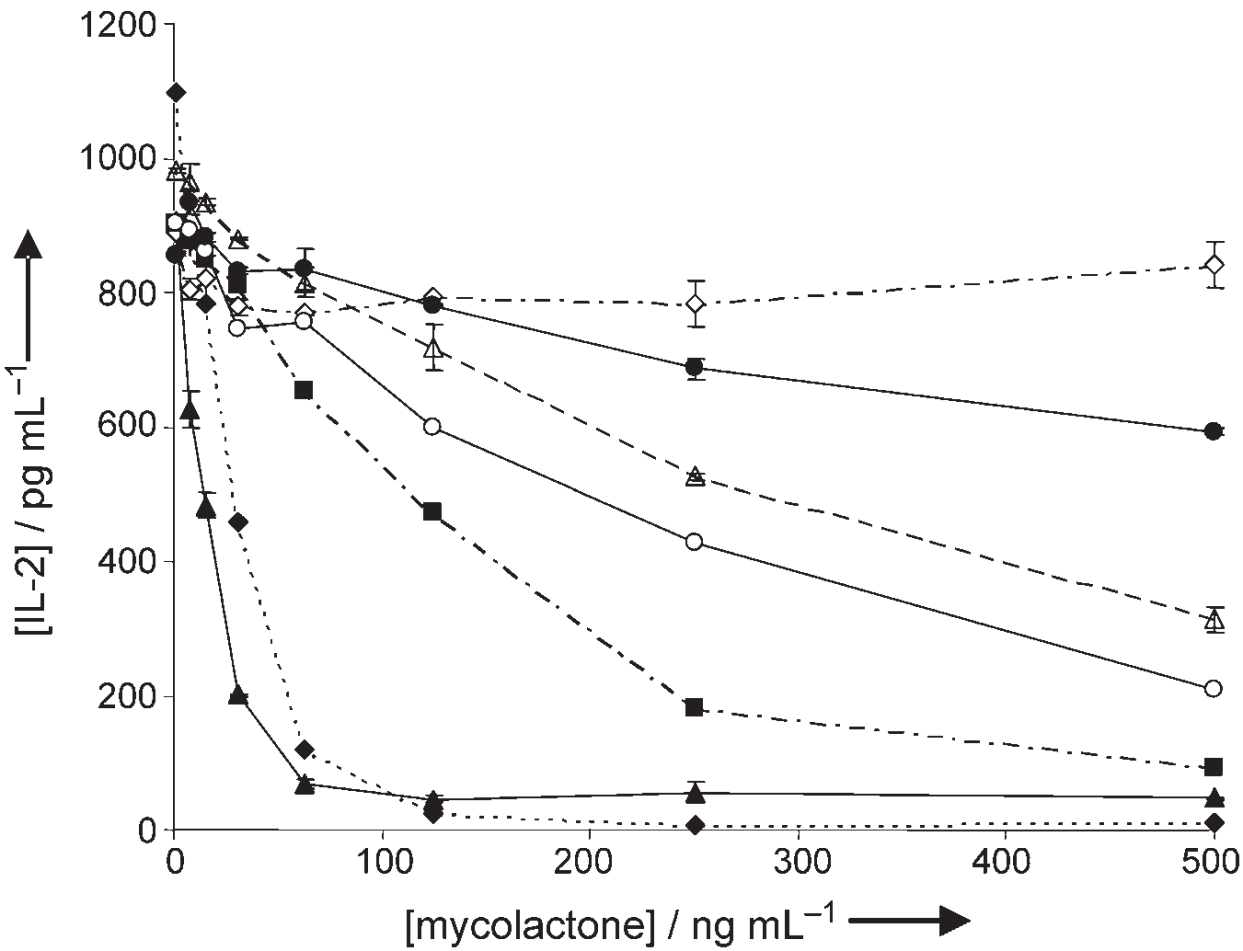
Differential immunosuppressive activity of mycolactone variants. IL-2 concentrations were measured in culture supernatants of Jurkat T cells activated after treatment with mycolactones or mycolactone mixtures: ▴: AB, ♦: AB+30 % C (AB+C), ▵: C+30 % AB (C+AB), ▪: E, ○: F, •: G, or ◊: ethanol as solvent control. The data are the mean ±SD of duplicate measurements, and are representative of two independent experiments.

## Experimental Section

**Microbiological methods**: The plasmid pJKD2888 was constructed in *Escherichia coli* DH10B by ligating a 1828 bp PCR fragment that included the 1314 bp *cyp140A7* (MUP_053) gene and 514 bp of upstream sequence into the unique *Xba*I site of the mycobacteria/*E. coli* shuttle vector pMV261.^19^ *M. ulcerans* strain Agy99 was used as a source of template DNA, and the PCR was primed by using the oligonucleotides MUP450F 5′-ggtctagatacacccttaccgcgacagt-3′ and MUP450R 5′-ggtctagaagccagtggcattgtcagat-3′. Electrocompetent *Mycobacterium marinum* DL045 cells were prepared as described^20^ and transformed with 5 μg of either pJKD2888 or the empty vector. Electroporated bacteria were incubated overnight in Middlebrook 7H9 medium (1 mL) at 30 °C then plated onto Middlebrook 7H10 agar, containing kanamycin (25 μg mL^−1^). Incubation at 30 °C was continued for 14 days. Kanamycin-resistant colonies were selected and subcultured into 7H9 medium (500 mL) and incubated at 30 °C for a further 14 days. Mycobacteria were confirmed as harbouring the correct plasmids by back transformation to *E. coli* followed by PCR, restriction enzyme digestion and DNA sequencing of the recovered plasmid. The preparation of cell extracts for mycolactone analysis was performed as described.^10^

**LC-MS analysis**: LC-MS and LC-MS/MS analyses were carried out on a Finnigan LCQ instrument as previously described.^10^ High-resolution MS and MS/MS analyses were performed on a LTQ Orbitrap instrument (Thermo Scientific, San Jose, CA, USA) and on a BioApex II (4.7 Tesla) FTICR mass spectrometer (Bruker Daltonics, Billerica, MA, USA).

**Assay of biological activity**: The human T-cell line Jurkat (subclone E6.1) was cultured in RPMI medium with 10 % foetal calf serum, l-glutamine (2 mm), penicillin (100 IU mL^−1^) and streptomycin (100 μg mL^−1^). Cells were incubated in microtitre plates (5×10^5^ cells per mL) with mycolactone preparations (0 to 500 ng mL^−1^) for 6 h, then activated with PMA (25 ng mL^−1^) and ionomycin (500 ng mL^−1^; both from Calbiochem, La Jolla, CA) for 16 h. Culture supernatants were assayed for IL-2 by ELISA (R&D, Minneapolis, MN).
